# Automatic materials characterization from infrared spectra using convolutional neural networks†

**DOI:** 10.1039/d2sc05892h

**Published:** 2023-02-23

**Authors:** Guwon Jung, Son Gyo Jung, Jacqueline M. Cole

**Affiliations:** a Department of Physics, Cavendish Laboratory, University of Cambridge J. J. Thomson Avenue Cambridge CB3 0HE UK jmc61@cam.ac.uk; b Scientific Computing Department, Science and Technology Facilities Council Didcot OX11 0FA UK; c Research Complex at Harwell, Rutherford Appleton Laboratory Didcot Oxfordshire OX11 OQX UK; d ISIS Neutron and Muon Source, STFC Rutherford Appleton Laboratory Harwell Science and Innovation Campus, Didcot OX11 0QX UK

## Abstract

Infrared spectroscopy is a ubiquitous technique used to characterize unknown materials in the form of solids, liquids, or gases by identifying the constituent functional groups of molecules through the analysis of obtained spectra. The conventional method of spectral interpretation demands the expertise of a trained spectroscopist as it is tedious and prone to error, particularly for complex molecules which have poor representation in the literature. Herein, we present a novel method for automatically identifying functional groups in molecules given the corresponding infrared spectra, which requires no recourse to database-searching, rule-based, or peak-matching methods. Our model employs convolutional neural networks that are capable of successfully classifying 37 functional groups which have been trained and tested on 50 936 infrared spectra and 30 611 unique molecules. Our approach demonstrates its practical relevance in the autonomous analytical identification of functional groups in organic molecules from infrared spectra.

## Abstract 2

Infrared spectroscopy is a technique used to characterize unknown materials by identifying the constituent functional groups of molecules through the analysis of obtained spectra. This analysis has now been automated using artificial intelligence.

## Graphical abstract

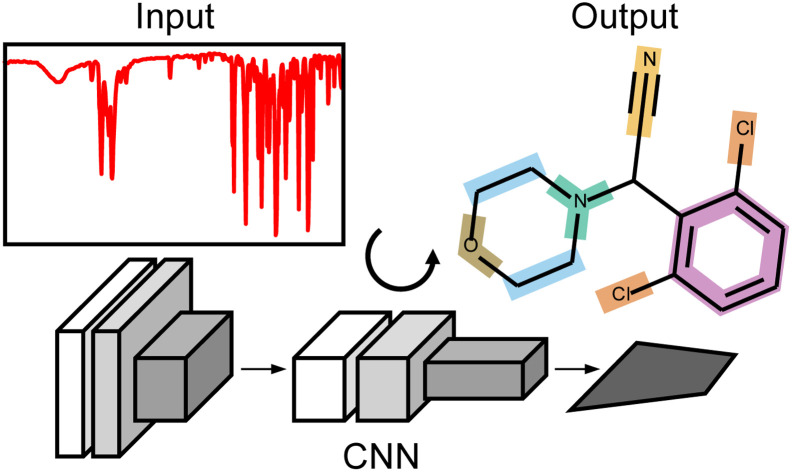

## Introduction

Molecules can be divided into functional groups, which are specific groups of atoms bonded in certain arrangements that together exhibit their own characteristic properties. The same functional group will have the same or similar physical and chemical properties regardless of the molecule of which it is a part.^1^ The presence of certain functional groups can affect the physical properties of a molecule, including its boiling point,^2^ solubility,^3^ and viscosity.^4^ Since the chemical properties of a functional group can modify the reactivity of other functional groups nearby, the technique of retro-synthetic analysis can be implemented to solve problems in the planning of organic syntheses.^5,6^ Inversely, structure elucidation of molecules can be achieved by observing the characteristic properties of constituent functional groups in unknown molecules. This applicable use of functional groups exemplifies the undeniable importance of identifying them.

Infrared (IR) spectroscopy exploits the relationship between the vibrational frequencies of chemical bonds in functional groups of molecules that absorb IR light and the structure of a molecule in order to determine the chemical identity of a compound. These absorptions occur at resonant frequencies where the vibrational frequency of a specific bond matches the frequency of the absorbed radiation. An IR spectrometer is the instrument used to realize the type of data, producing an IR spectrum by observing the differences in the intensities of incident and transmitted electromagnetic rays across a frequency range of around 400–4000 cm^−1^.^7^ The measured change in the intensities is illustrated in a graph of absorbance (or transmittance) against frequency (or wavelength) to form an IR spectrum from which the presence of a given functional group in a sample molecule can be deduced by analyzing its characteristic patterns of peak frequencies.

IR spectra can be manually interpreted by a spectroscopist, albeit there are factors that can make this process particularly challenging and inefficient. There may be significant shifts in the characteristic peak frequencies of certain functional groups from their archetypal ranges due to changes in their structural environment as a result of various physical and chemical factors that influence the components of the sample. Such factors include solvent effects, chemical reactions, and tautomerism. The region between 400–1500 cm^−1^, the fingerprint region, typically contains a vast number of peaks, and there is a high degree of overlap between spectral bands of molecular vibrations, making it difficult for a spectroscopist to resolve individual peaks manually. Accordingly, manual interpretation of IR spectra is usually performed only in the range 1500–4000 cm^−1^. This means that the unique information held within the fingerprint region of an IR spectrum, which can in principle be used to distinguish between different molecules, will be ignored. Even without the interpretation of the fingerprint region, the manual examination of many spectra is time-consuming, and it calls for the skills of a trained spectroscopist, who is vulnerable to human bias and error, especially in the case of complex and multifunctional compounds. The clear limitations in manual interpretation of IR spectra to identify functional groups call for other characterization techniques to be employed, such as computer-aided spectral interpretation methods.

During the 1990s, a number of neural-network approaches to spectral interpretation were explored.^8–11^ In comparison to current standards, the systems created were constrained by several factors including the size of the published training data, the complexity of the neural-network architecture explorable owing to low processing power, and a dearth in machine-learning techniques that were available at that time. The effects of these limitations are directly reflected in the performance of these systems. Given these historical limitations of neural networks, library-search^12–17^ methods and expert^18–22^ systems have evolved as the most common spectral interpretation frameworks, and they remain dominant in the *modus operando* of available IR data-analysis software to date.

The library-search method compares each IR spectrum from a collection of referenced IR spectra with an IR spectrum of an unknown molecule. Because IR spectra are subject to noise and to experimental conditions, exact matches cannot be expected. Therefore, the algorithm usually computes a degree of similarity between the IR spectra of the unknown and the database of reference IR spectra to construct a list of compounds, ranked by decreasing order of spectral similarity. The similarity metrics used in commercially available library-search algorithms include: the correlation coefficient, which is calculated from the absorbance values or the mean-centered absorbances of the spectra; the Euclidean distance; and the absolute absorbance differences.^23^ The library-search method has two major drawbacks. First, the associated software needs a large database of high-quality reference IR spectra, and it cannot identify the unknown compound if it is not stored in the database. However, it seems reasonable to think that similar spectra retrieved correspond to compounds whose molecular structure or key functional groups of molecular structure are more or less similar to the unknown. Second, a powerful computing system is necessary to be able to quickly search this large database.

Expert systems exploit the dependence of IR-spectral peak frequencies upon functional-group characteristics of a molecule to define its structural fragments. The first systems followed closely the classical ways that chemists identify unknown compounds where a table-driven classification procedure was applied for fast interpretation of IR spectra to receive a list of substructures that were probably a part of an analyzed compound; these substructures then need to be pieced together by the data analyst to identify the parent molecule of the sample compound. In most systems, knowledge about spectrum-structure correlation is formulated as interpretation rules that indicate the presence or absence of certain molecular fragments, when applied to a spectrum of an unknown structure. These rules can be based on the empirical examination of many reference IR spectra of known compounds or generated from a library of IR spectra *via* neural networks or the application of statistical algorithms. The major limitation of such expert systems is the nature of the knowledge concerned, in that the spectrum-structure correlations taken from the literature are directly stated by human experts or are extracted automatically from spectral databases which have been accumulated by the same type of experts. Affolter *et al.*^24^ tested a set of normal-frequency ranges for large fragments of organic molecules that had been tabulated from a manually curated database of IR spectra. They showed that the spectrum-structure correlation tables cannot be reliably used for automatic spectral interpretation, as the direct use of tabulated normal-frequency ranges taken from the literature leads to a high percentage of cases in which at least one of the ranges lacked any peaks. These expert systems also involve acquiring and manipulating large volumes of information or data. Taken together, these limitations call for the development of more sophisticated methods for the interpretation of IR spectra.

There have been recent developments in the application of machine learning to IR spectral interpretation, most of which are focused on a specific group of materials. For example, Michel *et al.*^25^ employed support-vector machines (SVMs) to develop a robust and reproducible method to identify six consumer plastics, in an attempt to understand the environmental fate and transport of macro- and micro-plastic debris. Chen *et al.*^26^ combined machine-learning algorithms and mid-IR spectroscopy to identify patients with glioma, which has a low cure rate and a high mortality rate. They first used principal-component-analysis algorithms to reduce data dimensionality, after which an SVM, a backpropagation neural network, and a decision-tree model were established. They found that the SVM model had the highest classification accuracy. Fine *et al.*^27^ successfully applied a multilayer perceptron (MLP) to classify 16 functional groups of a broader set of materials. MLPs have fully connected layers, where each node in one layer is connected to all nodes in the succeeding layer, making them prone to overfitting data. The authors made use of IR spectra and the corresponding mass spectra of 7000 compounds. Wang *et al.*^28^ also constructed SVM models to infer the presence or absence of 16 functional groups using a total set of 823 compounds. Their results were evaluated by a variety of indices and compared favorably with those obtained by using artificial-neural-networks (ANN) methods.

In this work, we demonstrate that convolutional neural networks (CNNs) can be used to identify 37 functional groups of organic molecules from a dataset of IR spectra that spans 30 000 unique compounds. CNNs expand upon ANNs by sliding convolution kernels (or filters), which have a shared-weight architecture, across input features and yield translational equivariant responses known as feature maps.^29^ The initial filters are embossed with small and simple patterns that are used to assemble increasingly complex patterns, taking advantage of the hierarchical patterns within the data. This significantly increases the number of pixels handled per neuron compared to an MLP, reducing the connectivity and complexity of the network architecture without compromising its performance. Another major advantage of using CNNs is that there is an independence from prior knowledge and human intervention since they require little or no pre-processing, thereby enabling the network to learn to optimize the filters through automated learning for feature extraction. We show that a one-dimensional CNN can be implemented to develop an automated IR spectral interpretation system capable of identifying 37 functional groups with a high degree of confidence.

## Results and discussion

### CNN architecture

**Fig. 1:**
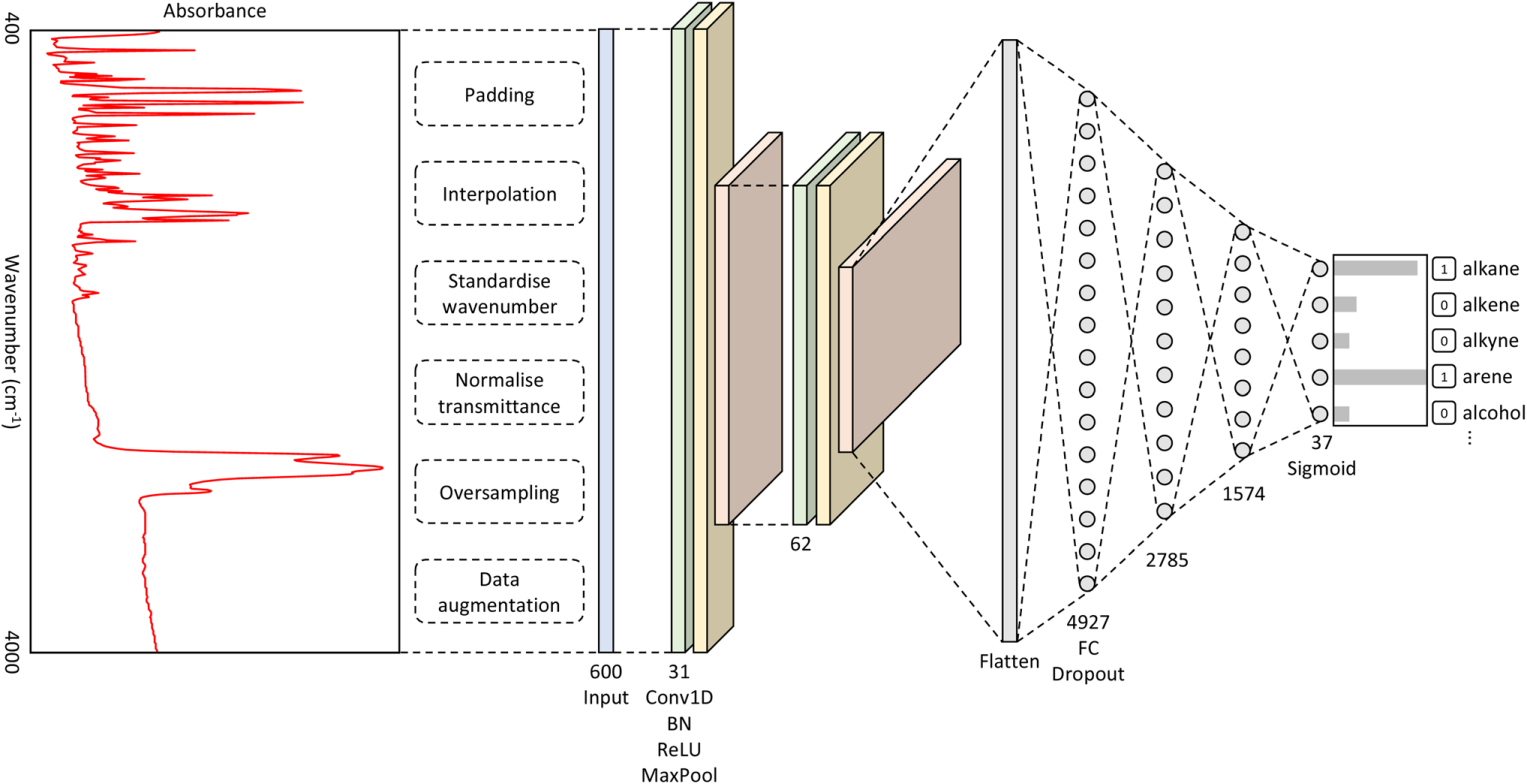
A diagram showing the architecture of our CNN model. The network takes in an input vector of length 600, which is the intensity values for each wavenumber. The convolutional layers consist of repeating Conv1D, BatchNorm (BN), ReLU, and MaxPool operations. The fully connected (FC) layers are followed by the dropout operation. The output layer comprises 37 outputs, representing the number of functional groups; the sigmoid activation function was used for this layer.

The best-performing hyperparameters were used to build our CNN model, as shown in Fig. 1. It takes as input a spectrum of an unknown material and outputs a classification of functional groups that pertain to the distinctive spectral features. The full details of the methods that were used to produce this overall CNN architecture can be found in the ESI.†

### Original *versus* extended model performance

We initially defined the 22 most common functional groups to train the original model; an extended model was then trained using all 37 functional groups, thereby incorporating less common functional groups into the model. We trained our model to be a multi-label classifier in order to predict all functional groups simultaneously.

**Fig. 2:**
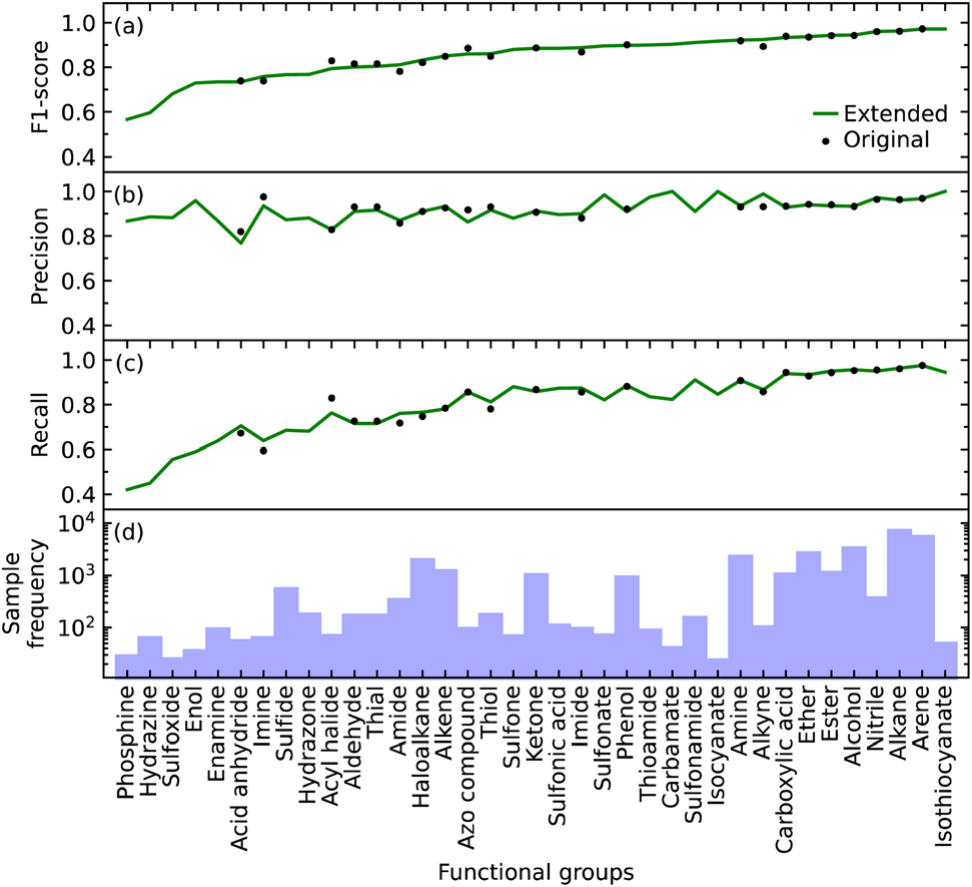
The (a) F1 score, (b) precision, and (c) recall for the identification of each functional group in the original model and the extended model, which include 22 and 37 functional groups, respectively; (d) sample frequency of each functional group.

We found that the inclusion of the additional 15 functional groups did not hinder the performance of our model in predicting the original list of functional groups, as shown in Fig. 2b–d. The largest increase and decrease in the F1 scores, when implementing the extended list instead of the original list, were +0.031 and −0.034 for alkyne and acyl halide groups, respectively. These small changes in the F1 score indicate that the original and extended models are equally fit for purpose. They furthermore suggest that even more functional groups could, in fact, be introduced into the training of such a model, pending the availability of additional IR-spectral data on molecules that contain many instances of other functional groups. Such an expansion would stand to offer a more precise structural characterization of materials while maintaining model performance. For example, these other functional groups could be more complex molecular fragments, such as heterocyclic, aromatic rings, or subdivisions of functional groups. Both approaches would bring our model one step closer to full autonomy *via* minimal manual intervention. While the lack of suitable datasets renders this potential expansion beyond the scope of this work, the well-performing and open-source nature of our code base offers the tools for readers to create such an expansion. However, this comes with the caveat that the addition of certain molecular fragments, such as described above, can prove to be difficult. Due to vibrational coupling, spectral features can and frequently are very complex, adding to the complexity of the overall spectrum.

### Relative abilities of the extended model to identify each functional group

The F1 score of all but a few of the functional groups ranges between 0.57 and 0.97, with values being above 0.73 except for those pertaining to the phosphine, hydrazine, and sulfoxide groups; see Fig. 2a. The precision values are consistently high, ranging from 0.77 to 1.00, and all but one functional group (acid anhydride) are greater than 0.83; see Fig. 2b. However, the range of recall values was relatively broad, extending as low as 0.42 and as high as 0.97; see Fig. 2c. There does not seem to be a clear reason as to why the recall values associated with certain functional groups are considerably lower than others, besides their lack of coverage in the training data *cf.* the numbers of samples for each of the 37 functional groups is displayed in Fig. 2d. However, our model could identify certain functional groups with low coverage very well. Nonetheless, there are exceptions to consider; for example, isothiocyanate had the highest F1 score of 0.97 with only 216 samples, while arene and alkane had F1 scores of 0.97 and 0.96 with 23 657 and 31 201 samples, respectively. Hence, functional groups with fewer samples should not be left out of consideration simply because of the lack of coverage.

Overall, the original model had an average F1 score of 0.87 and a weighted average F1 score of 0.93. The extended model had an average F1 score of 0.85 and a weighted average F1 score of 0.93. Since the inclusion of more functional groups has a minimal effect on the performance of our model, we will solely use the extended model for further analyses.

### Multiple predictions

While it is important for our model to accurately predict the presence of a single functional group, a metric better suited to measure the performance of predicting all functional groups in a given molecule is required when considering the ultimate task of materials characterization. This is because the prediction of multiple functional groups in a single molecule poses a different optimization problem. To this end, we investigated how the EMR was affected for molecules with one through ten functional groups (maximum in our data) using the extended set of functional groups.

**Fig. 3:**
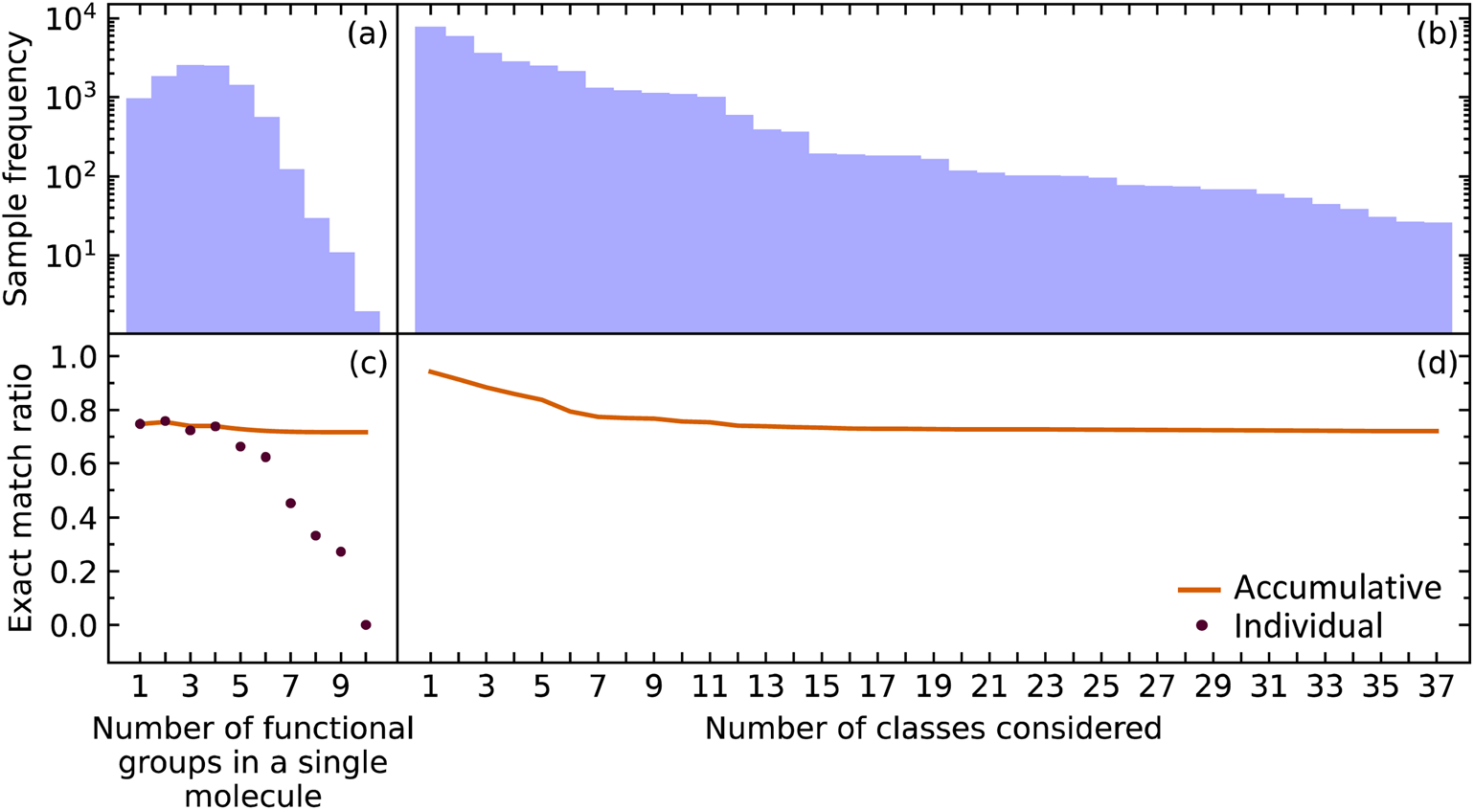
The (a) sample frequency and (b) exact match rate (EMR) for molecules with differing number of functional groups. The (c) sample frequency and (d) EMR for differing number of classes.

The EMR for individual EMR values as well as the accumulated EMR values is shown in Fig. 3. We hypothesized that an increase in the number of functional groups to be predicted would decrease the EMR. Although the individual EMR for higher numbers of functional groups in a single molecule was found to drop drastically after six functional groups per molecule, the sample frequency of these molecules also dropped. Therefore, it turns out that the effect on the accumulative EMR, which plateaus at around 0.72, is minimal. Since molecules with more than ten functional groups will rarely be characterized *via* FTIR spectroscopy, and that the sample frequency of the less common functional groups will be relatively low, it is reasonable to say that our model has an overall accumulated EMR of 0.72.

We also explored how our model performed while varying the number of functional groups that were considered. The order of functional groups considered was chosen from the most abundant to the least abundant functional groups; although, this order is essentially arbitrary because considering all 37 functional groups in any order would result in the same final EMR. As hypothesized, the EMR decreases as the number of classes predicted increases. Since most of the common functional groups in the organic chemistry domain have already been included in our model, the predictive classification of any additional functional groups would have fewer samples and its effect on the EMR would be negligible. The EMR for varying the number of functional groups in the predictive-classification task plateaus at around 0.72.

Given that EMR is a stringent metric, we conclude that the addition of further class labels to our classification model in order to predict more functional groups is possible without compromising its ability to predict other functional groups. In addition, the number of functional groups enclosed in a single molecule will not have a drastic effect on the EMR.

### Class imbalance

The class label for each functional group carries a positive or negative sign according to whether the model classifies the group as being present in a subject molecule or not. Given that there are typically many more options for functional groups (class labels) than the number of such groups which are present in a given molecule, there is a large imbalance between the positive and negative class labels in which the negatives dominate. Therefore, we also evaluated our model by focusing on the positive class labels.

**Fig. 4:**
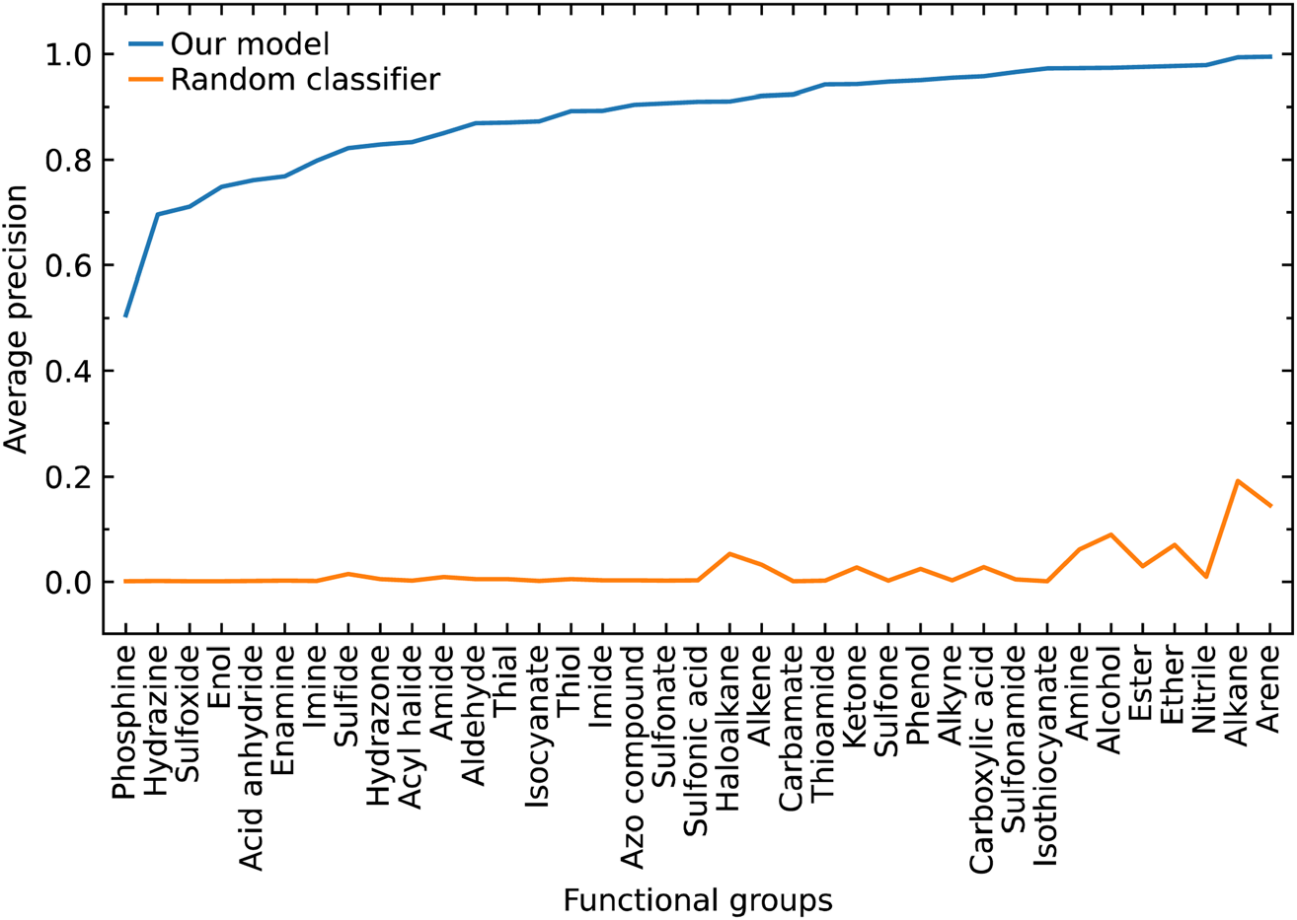
Average precision of our predictive classification model shown against that of a random classifier based on the proportion of the positive signs in each class label that represents a given functional group.

We calculated the AP to summarize PR curves as the weighted mean of precisions acquired at each threshold, with the increase in recall from the previous threshold used as the weight. Here, threshold refers to the classification threshold used to convert predicted probabilities to class labels in order to indicate whether or not a functional group is present. The AP for the correct classification of each functional group is shown in Fig. 4. It shows if our model can accurately identify all relevant class labels for functional groups as positives without mistakenly labeling too many negatives as positives. Thus, AP is higher when our model can correctly handle positive class-label assignments for each functional group. The AP of a random classifier is equal to the true proportion of positive class labels, and the AP of a perfect classifier is 1.0. Fig. 4 shows that our model is efficient at identifying positive class labels even when the AP of a random classifier is close to zero. The mean average precision (mAP) of our model for the classification of 37 functional groups is 0.88.

**Fig. 5:**
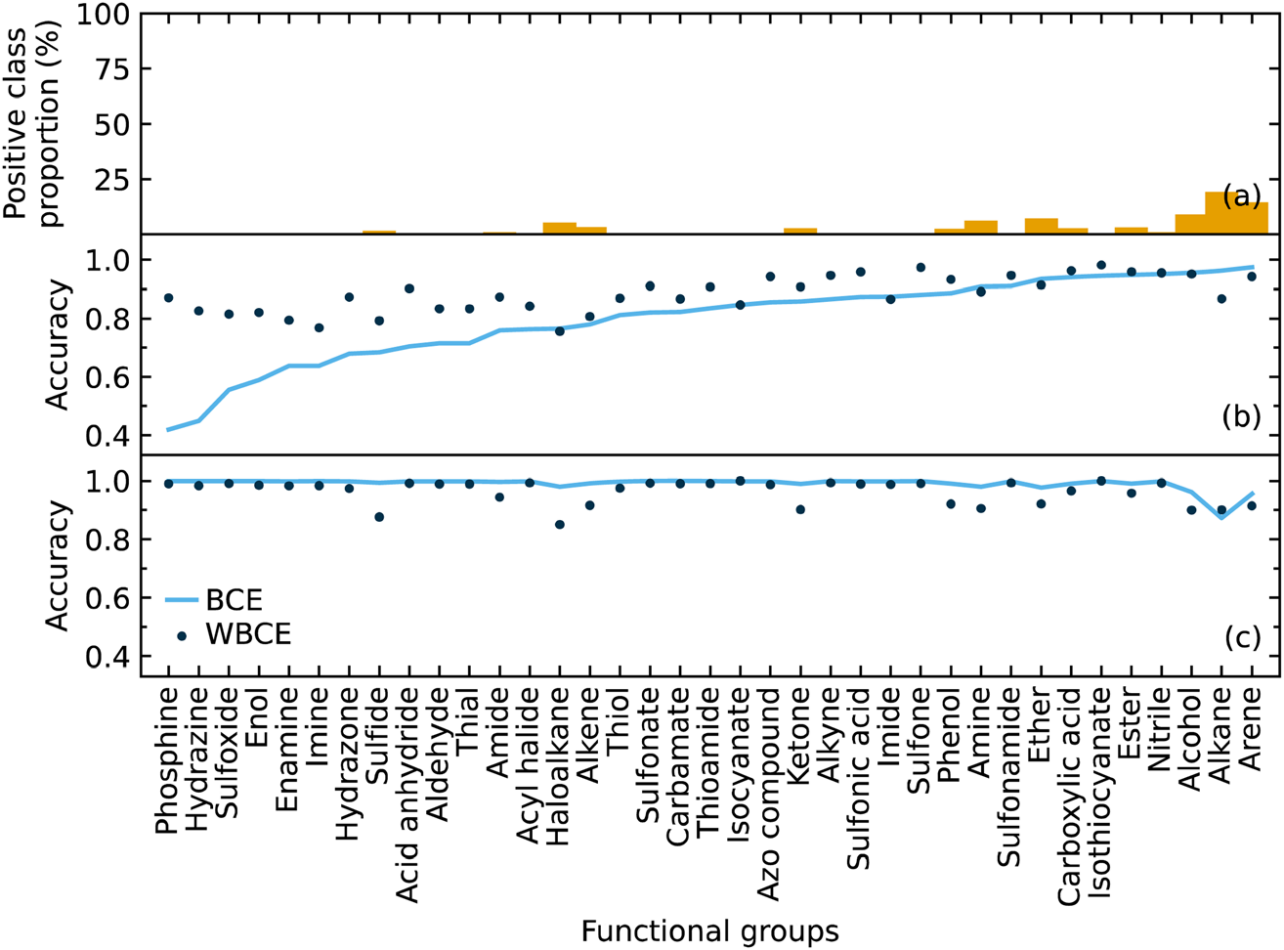
The (a) proportion of the positive class labels for each functional group. The accuracies for the (b) presence and (c) absence of functional groups using binary cross-entropy and weighted binary cross-entropy as the loss function.

We implemented weighted binary cross-entropy (WBCE) as the loss function, as was defined in eqn (S7),† in order to improve the prediction accuracy of the minorities. We calculated these accuracies for the presence and absence of functional groups separately rather than calculate the overall accuracy for each class label in order to see the effects of the WBCE function more clearly. Fig. 5 shows the average accuracy for the presence of functional groups, while implementing binary cross-entropy (BCE) and WBCE, was 0.80 and 0.88, respectively. The average accuracy for the absence of functional groups, while implementing BCE and WBCE, was 0.99 and 0.96, respectively.

We managed to significantly improve the accuracy for the predictions of the minorities with only a slight decrease in the accuracy for the predictions of the majorities. However, owing to the huge class imbalance, the slight decline in the accuracies for the classification of minorities notably affected the overall accuracy, which dropped from 0.99 to 0.96. Therefore, the WBCE was not implemented in the final model, but a separate model was made available; see software and availability section for details.

### Data augmentation and oversampling

As a CNN is a data-hungry model, we examined how oversampling and three different methods of augmentation (horizontal shift, vertical noise, and linear combination) would affect the performance of our CNN model. Oversampling was used as a control method to compare against the augmentation methods, by augmenting the same sampled data. We varied the amount of sampling from 25% to 100% of the training data, in steps of 25%. We also compared the results with the performance of our model trained only on the original data.

**Fig. 6:**
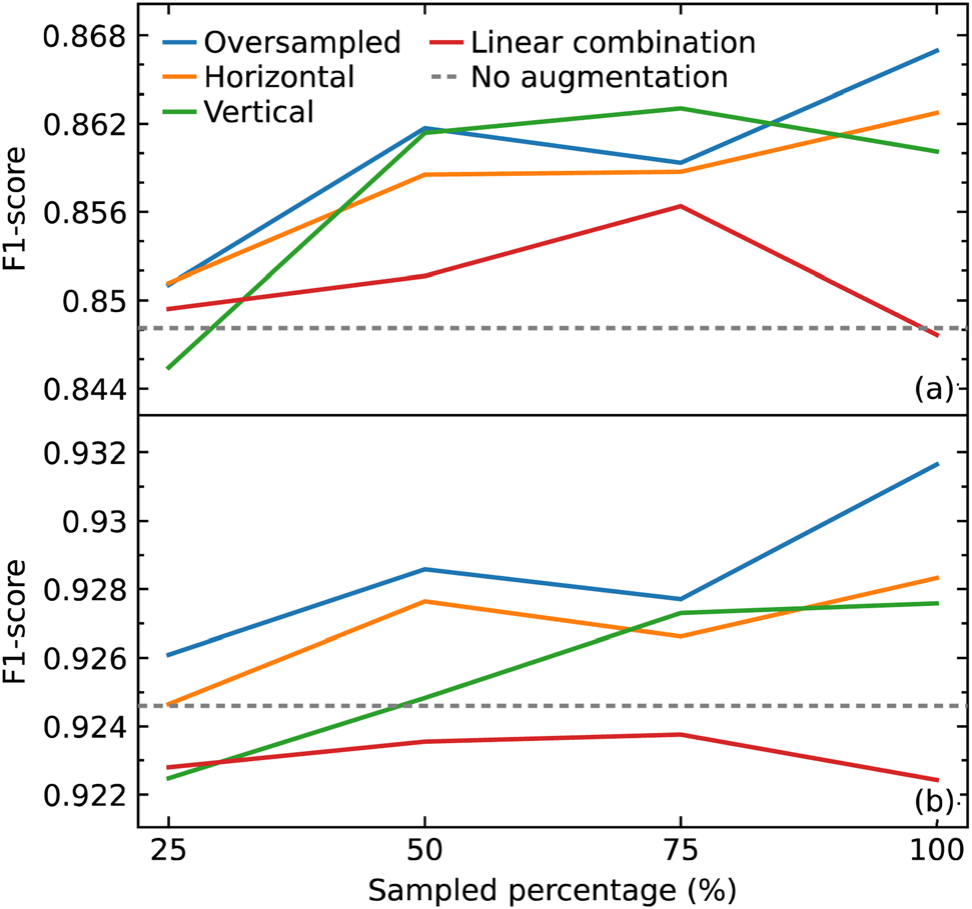
(a) Macro average F1 scores (b) and weighted average F1 scores for models trained with addition of oversampled data and augmented (horizontal shift, vertical noise, linear combination) data compared with that trained without the extra data.

We hypothesized that all the augmentation methods and oversampling of the training data would generally improve the performance of the model. While this was true when performing and evaluation with the macro average F1 score, except in two cases (25% sampling of vertical augmentation and 100% sampling of linear combination as shown in Fig. 6a), this is not the case when evaluating the effect of these data treatments with weighted average F1 score, as shown in Fig. 6b. The latter is more meaningful as it represents the number of true instances for each class label by altering ‘macro’ to account for class label imbalance.

All sampling proportions of the data augmentation by linear combination resulted in F1 scores that were lower than those without this data treatment. A simple explanation could be the highly sensitive nature of IR spectra, where two spectra of the same compounds can show significant differences in the peak intensities, shape, and frequency position (within a certain range) depending on various factors. As a result, depending on the coefficients selected, erroneous IR-spectral peak shapes might be created when two IR spectra are linearly mixed.

The horizontal shift of the spectrum is another form of augmentation that we investigated. Translational invariance is attained as a result of the max-pooling operation within our CNN model architecture. The max-pooling operation replaces the output of a convolutional layer at a specific position with the maximum of neighboring outputs. As a result, even if we adjust the inputs to the convolutional layer slightly, the values of most of the pooled outputs should remain unchanged. This indicates that even if the inputs to the convolutional layer are translated, the predictive output of the CNN model will still be able to recognize the class of functional group to which it belongs. As a consequence, we hypothesized that augmenting the data with horizontal translations would improve the performance of our CNN model, as demonstrated in Fig. 6a and b.

When noise was added to a spectrum, the magnitude of the noise at a certain point was determined by the intensity value at that point. This method was employed to introduce noise with a corresponding level of magnitude throughout the spectrum. The addition of noise did not improve the performance of the model. One possible explanation for the lack of effectiveness of augmentation by adding noise is that it may have altered the shape of the peaks in a way that the CNN model would recognize the pattern to be completely different to the original rather than one with slight variation.

With the exception of augmentation by linear combination, the general trend of the augmentation methods may appear to be an uptrend as the sample size increases. When compared to oversampling, however, the augmentation methods did not enhance the performance of the model, considering that the same oversampled data were augmented. The best improvement was achieved by oversampling 100% of the training data where the F1 score improved from 0.926 to 0.932. Overall, the improvements gained in the performance of the model from all augmentation methods and oversampling turned out to be insignificant.

### Optimal threshold tuning

One of the ways that we used to try to improve the performance of our model for the classification of functional groups was *via* optimal-threshold tuning. The optimal thresholds, which were used to convert probability predictions into crisp class labels, were computed based on the training dataset, following the training of the model. To begin, a line plot of recall (*x*-axis) against precision (*y*-axis) was produced in ascending order of the thresholds. Finding the threshold that results in the best balance between precision and recall is equivalent to optimizing for the F1 score that summarizes the harmonic mean of both metrics. We took the naive approach of calculating the F1 score for each threshold, then locating the threshold where the corresponding F1 score was the largest.

When employing the calculated optimal thresholds (Table 1) for interpreting the predicted probabilities of all functional groups, the average F1 score and the weighted average F1 score were 0.85 and 0.93, respectively. As mentioned in Section 3.1, when 0.5 was used as the threshold, we obtained the same average F1 score and weighted average F1 score. As there were no differences between the sets of F1 scores when applying the optimal thresholds and when using 0.5 for all interpretations, we used the latter for simplicity.

**Table 1:** Calculated optimal thresholds used to classify probability predictions into definite class labels for each functional group

Functional group	Optimal threshold
Alkane	0.466994
Alkene	0.326576
Alkyne	0.604118
Arene	0.658939
Haloalkane	0.282561
Alcohol	0.480895
Aldehyde	0.852833
Ketone	0.538769
Carboxylic acid	0.603919
Acid anhydride	0.785734
Acyl halide	0.342643
Ester	0.590797
Ether	0.410823
Amine	0.476953
Amide	0.503042
Nitrile	0.705652
Imide	0.682684
Imine	0.303812
Azo compound	0.165843
Thiol	0.497317
Thial	0.850316
Phenol	0.625987
Enol	0.857550
Sulfone	0.897004
Sulfonic acid	0.866157
Hydrazine	0.389779
Enamine	0.714258
Isocyanate	0.109453
Isothiocyanate	0.326955
Phosphine	0.471990
Sulfonamide	0.149206
Sulfonate	0.879403
Sulfoxide	0.530636
Thioamide	0.731061
Hydrazone	0.519286
Carbamate	0.399003
Sulfide	0.443587

### Demonstration of the model

**Fig. 7:**
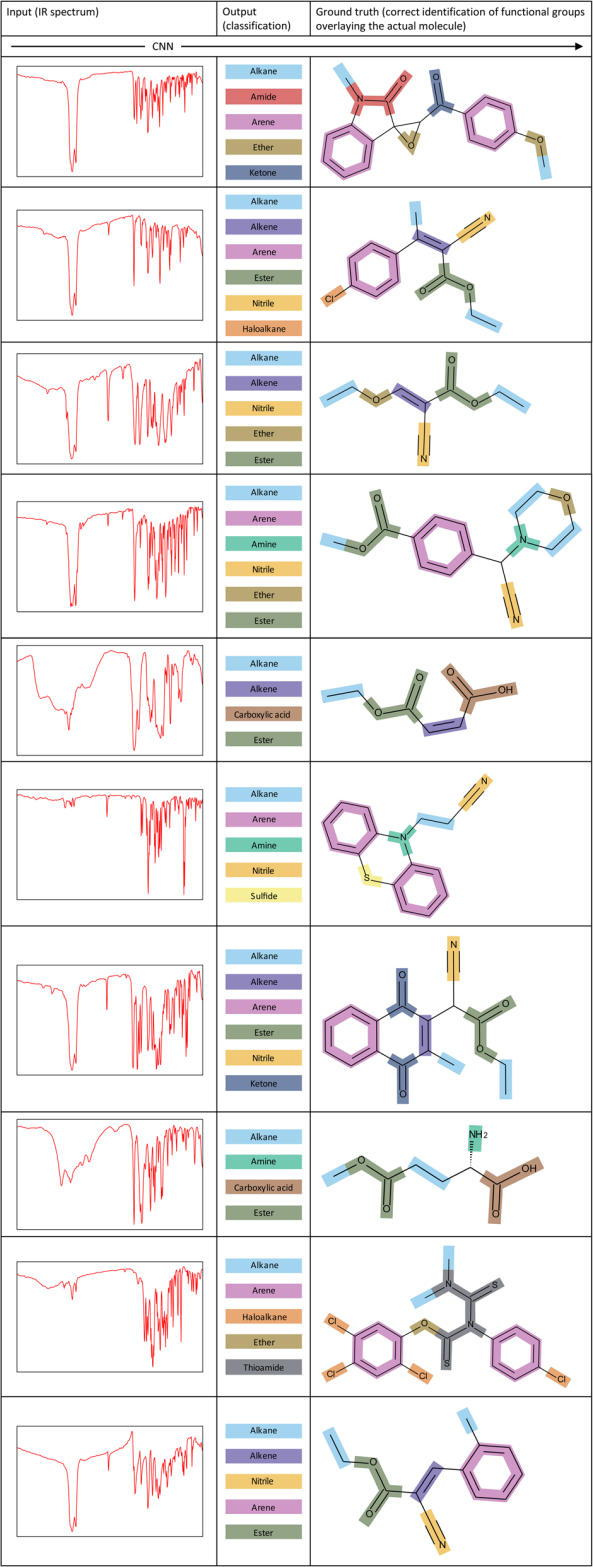
Examples of inputs and the corresponding outputs to and from our CNN model. The correct identification of functional groups overlaying the actual molecule is shown for each example.

To showcase the ability of our model more clearly, Fig. 7 illustrates several examples of corresponding inputs and outputs to and from our CNN model. The inputs are the IR spectra of the unknown molecules, and the outputs are the functional groups that are predicted to be present in the molecule. We also depict the correct identification of functional groups on the actual molecule to aid in interpretation of the output predictions.

## Conclusions

Most functional groups have the same or comparable innate qualities regardless of the molecule of which they are a part. When identifying or predicting the structure and properties of molecules, information regarding the existence of functional groups, which may be acquired by interpreting the corresponding spectrum, is critical. The conventional method of spectral interpretation requires the skills of an expert spectroscopist and can take a long time to interpret. We have presented a novel spectral interpretation approach that uses convolutional neural networks (CNNs) to identify spectral features in a spectrum, which are then analyzed to predict the presence of distinct functional groups. Our model was trained and validated on a huge collection of infrared (IR) spectra which includes a wide range of functional groups that work together to deliver a complete spectral interpretation. Our approach makes it simple to analyze organic compounds because of its autonomous nature. A range of case studies are used to demonstrate the practical application of our predictive CNN model. We conclude that CNNs are effective at identifying IR spectral features and are transferable to a chemical-identification application.

## Data availability

We have made the code for scraping the two data sources, pre-processing data, hyperparameter optimization, model training, optimal-threshold tuning, data augmentation, and the trained models used in this study is available at: https://github.com/gj475/irchracterizationcnn. The data sets used in this study are available from the NIST Chemistry WebBook (https://webbook.nist.gov) and the National Institute of Advanced Science and Technology, SDBS Web (https://sdbs.db.aist.go.jp).

## Author contributions

The overall idea was conceived by J. M. C. The study was designed by G. J. and J. M. C. Under the guidance of J. M. C. and G. J. created the workflow, performed data gathering, data pre-processing, data augmentation, hyperparameter optimization, model training, optimal-threshold tuning, and analysis. S. G. J. contributed to hyperparameter optimization and determining the best thresholds for interpreting expected probabilities. G. J. drafted the manuscript with the assistance of J. M. C. The final manuscript was read and approved by all authors.

## Conflicts of interest

The authors declare no competing financial interests.

## Supplementary Material

SC-014-D2SC05892H-s001
