# Supplementary material for: Automatic materials characterization from infrared spectra using convolutional neural networks
Source: Chem Sci. 2023 Feb 23;14(13):3600–9. doi: 10.1039/d2sc05892h (PMC10055241; doi:10.1039/d2sc05892h)
Supplement: SC-014-D2SC05892H-s001 [file SC-014-D2SC05892H-s001.pdf]

Electronic Supplementary Material (ESI) for Chemical Science.
This journal is © The Royal Society of Chemistry 2023

Supplementary information

Automatic materials characterization from infrared spectra using convolutional neural networks

Guwon Jung,[1,2,3] Son Gyo Jung[1,3,4], Jacqueline M. Cole[1,3,4,*]

* Corresponding author

[1] Cavendish Laboratory, Department of Physics, University of Cambridge, J. J. Thomson Avenue, Cambridge, CB3 0HE, UK
E-mail: jmc61@cam.ac.uk

[2] Scientific Computing Department, Science and Technology Facilities Council, Didcot, OX11 0FA, UK

[3] Research Complex at Harwell, Rutherford Appleton Laboratory, Didcot, Oxfordshire OX11 0QX, U.K

[4] ISIS Neutron and Muon Source, STFC Rutherford Appleton Laboratory, Harwell Science and Innovation Campus, Didcot, OX11 0QX, UK

Table of contents

Methods 3
Data collection 3
Choice of functional groups 4
Hyperparameter tuning 7
CNN architecture 9
CNN training 11
Evaluation metrics 13
Supplementary tables 15
References 15

Methods

Data collection

We obtained data from the National Institute of Advanced Industrial Science and Technology (AIST)[1] which comprises spectra of materials that were acquired in one of four different types of condensed-phase sample environment: KBr disc, Nujol mull, single-component liquid film, and CCl_4 solution. The spectra were downloaded as image files which were subsequently digitalized using pixel mapping. This entailed detecting vertical and horizontal lines that form a rectangle of a specific size in order to identify the region of interest (ROI). Raster scanning was used to inspect each pixel in the ROI, saving the x and y coordinates of pixels whose colors exceed a certain threshold of red-green-blue (RGB) values. The extracted spectra had a resolution of 4 cm^{-1} in the low wavenumber range (400-2000 cm^{-1}), while the resolution in the high wavenumber range (2000-4000 cm^{-1}) was 8 cm^{-1}. AIST data contained only transmittance spectra. We also gathered data from the National Institute of Standards and Technology (NIST)[2] that contained gas-phase spectra. The spectra from NIST were obtained as JCAMP-DX files, which had a resolution of 2 cm^{-1}. NIST data contained both transmittance and absorbance spectra.

It was necessary to normalize these spectra from different sources to present them on the same scale. All absorbance spectra from the NIST database were converted to transmittance spectra. The raw spectra from the two data sources were interpolated with first-order splines in order to obtain vectors with 600 normalized transmittance values (at 6 cm^{-1} intervals) across the frequency range of 400 cm^{-1} to 4000 cm^{-1}. A relatively low resolution was chosen

because CNNs could detect patterns in the data with reduced dimensionality. Some of the NIST spectra had gaps in the transmittance values where measurements were not taken over the entire range of 400-4000 cm^{-1}. AIST spectra collected in CCl_4 solution exhibited discontinuities in the 690-850 cm^{-1} and 1500-1600 cm^{-1} frequency ranges owing to heavy solvent absorption of the IR beam, which were filled during the interpolation step.

For each spectrum, the InChI string of its sample material was scraped. InChI was chosen over the alternate SMILES chemical-identity representation for this purpose because it intends to give a unique (or canonical) identifier for chemical structures, whereas SMILES strings are used for storage and interchange of chemical structures without a standard for generating canonical strings.[3]

Certain spectra from the data sources were removed from consideration by manual selection for various reasons. If spectra from either data source could not be linked to valid InChI strings, they were removed. This accounted for 3437 spectra that were not included. A selection of low-quality spectral images obtained from AIST were excluded, accounting for 2832 spectra. Some InChI strings were converted to MOL format incorrectly, resulting in the exclusion of 83 spectra. After this data pruning stage, the two sources of data afforded 50,936 IR spectra on 30,611 compounds with unique InChI strings.

Choice of functional groups

We chose 37 functional groups to enable our task of chemical identification from IR-spectra. This choice reflects the abundance of each functional group in the two dataset sources and

those that are commonly identified using FTIR spectroscopy. The most commonly characterized functional groups were used to train the machine-learning model that can draw insights from learned chemical patterns in the same manner that human chemists can do. It is worth adding that this model could be expanded in future work to adopt more abstract definitions of functional groups, e.g., if one wants to tailor the model to identify certain series of compounds. We eliminated any functional groups that had fewer than 130 samples within the dataset. These excluded functional groups are shown in Table S2. We defined and tested SMARTS strings for each of the functional groups, as shown in Table S1. For every sample, substructure matching was performed between a molecule constructed from its InChI string and each of the 37 molecules that had been constructed from SMARTS strings of the 37 functional groups using RDKit.[4] Through this process, labels were created to record the presence of the functional groups.

Table S1. Definition of SMARTS strings used to identify the presence of functional groups in each sample molecule. The prevalence of each functional group across the entire dataset is also displayed. *Functional groups that were included in the training of the original model.

Functional group	SMARTS string	Sample frequency
Acid anhydride*	[CX3](=[OX1])[OX2][CX3](=[OX1])	285
Acyl halide*	[CX3](=[OX1])[F,Cl,Br,I]	354
Alcohol*	[#6][OX2H]	18142
Aldehyde*	[CX3H1](=O)[#6,H]	928
Alkane*	[CX4;H3,H2]	39002
Alkene*	[CX3]=[CX3]	6593
Alkyne*	[CX2]#[CX2]	562
Amide*	[NX3][CX3](=[OX1])[#6]	1873
Amine*	[NX3;H2,H1,H0;!$(NC=O)]	12521
Arene*	[cX3]1[cX3][cX3][cX3][cX3][cX3]1	29571
Azo compound*	[#6][NX2]=[NX2][#6]	501
Carbamate	[NX3][CX3](=[OX1])[OX2H0]	239
Carboxylic acid*	[CX3](=O)[OX2H]	5282
Enamine	[NX3][CX3]=[CX3]	455
Enol	[OX2H][#6X3]=[#6]	157
Ester*	[#6][CX3](=O)[OX2H0][#6]	6614
Ether*	[OD2]([#6])[#6]	14708
Haloalkane*	[#6][F,Cl,Br,I]	10723
Hydrazine	[NX3][NX3]	343
Hydrazone	[NX3][NX2]=[#6]	784
Imide*	[CX3](=[OX1])[NX3][CX3](=[OX1])	536
Imine*	[$([CX3]([#6])[#6]),$([CX3H][#6])]=[$([NX2][#6]),$([NX2H])]	326
Isocyanate	[NX2]=[C]=[O]	131
Isothiocyanate	[NX2]=[C]=[S]	270
Ketone*	[#6][CX3](=O)[#6]	5441
Nitrile*	[NX1]#[CX2]	1993
Phenol*	[OX2H][cX3]:[c]	4796
Phosphine	[PX3]	153
Sulfide	[#16X2H0]	3034
Sulfonamide	[#16X4]([NX3])(=[OX1])(=[OX1])[#6]	777
Sulfonate	[#16X4](=[OX1])(=[OX1])([#6])[OX2H0]	313
Sulfone	[#16X4](=[OX1])(=[OX1])([#6])[#6]	376
Sulfonic acid	[#16X4](=[OX1])(=[OX1])([#6])[OX2H]	468
Sulfoxide	[#16X3]=[OX1]	137
Thial*	[CX3H1](=O)[#6,H]	928
Thioamide	[NX3][CX3]=[SX1]	321
Thiol*	[#16X2H]	955

Hyperparameter tuning

The collected IR-spectral data were split into five equal parts using stratified random sampling where one part was reserved as the test set and the remaining four parts were used to perform a four-fold cross validation of the hyperparameter tuning where one part acted as the validation set.

Since tuning the hyperparameters of a CNN is computationally expensive, we used Bayesian optimization for this process as described in Algorithm S1. We aimed to find the global optimum $\mathbf{x}^*$ of an unknown objective function f where $f(\mathbf{x})$ was evaluated for $\mathbf{x} \in \mathcal{X}$. That is,

$$\mathbf{x}^* = \arg\min_{\mathbf{x} \in \mathcal{X}} f(\mathbf{x}), \quad \text{(S1)}$$

where $\mathcal{X}$ is a hyperparameter space that contains discrete and continuous variables (integers and real numbers).

The objective function was approximated using Gaussian processes (GPs) as the surrogate model. Prior functions were defined using GPs, which were used to incorporate prior beliefs about the objective function. As the GP posterior is inexpensive to test, it was used to suggest points in the search space where sampling is likely to result in an improvement.

We applied *expected improvement* as the acquisition function in order to direct sampling to regions where an improvement over the current best observation is anticipated. This

direction is accomplished by balancing exploration and exploitation: sampling where the predictive uncertainty is large against sampling where the surrogate model predicts a high objective. Given that f' is the minimal value of f observed so far, expected improvement evaluates f at the point where f' is expected to improve the most. This is expressed by the following utility function:

$$u(\mathbf{x}) = \max(0, f' - f(\mathbf{x})). \tag{S2}$$

This means that if $f(\mathbf{x})$ is less than f', a reward equal to the improvement $f' - f(\mathbf{x})$ is received, and no reward otherwise. Hence, the expected improvement is the expected utility as a function of $\mathbf{x}$:

$$\alpha_{\mathrm{EI}}(\mathbf{x}) = \mathbb{E}[u(\mathbf{x}) \mid \mathbf{x}, D], \tag{S3}$$

where D are the samples drawn from f so far.

Algorithm S1. Bayesian Optimization

for n = 1, 2, ..., **do**
    select new $\mathbf{x}_{n+1}$ by optimizing acquisition function α:
        $\mathbf{x}_{n+1} = \arg\max_{\mathbf{x}} \alpha(\mathbf{x};\, D_n)$
    query objective function to obtain y_{n+1}
    update samples $D_{n+1} = \{D_n, (\mathbf{x}_{n+1}, y_{n+1})\}$
    update statistical model

A total of 50 evaluations were made for each of the folds. During each evaluation, validation loss was monitored and if no improvement was seen for five consecutive epochs, the learning rate was reduced by a factor of 10 until the minimum learning rate of 1×10^{-6} was reached.

Training was terminated once validation loss stopped improving for 10 consecutive epochs. During this process, we monitored the binary cross-entropy metric, which was minimized. The dimensions, corresponding search space, and the best performing hyperparameters, that were explored are shown in Table S2.

Table S2. The optimized hyperparameters, search space, and optimum values for our CNN model.

Dimension	Search space	Optimum
Number of convolutional layers	Low = 1, high = 5 (integer)	2
Number of filters	Low = 4, high = 32 (integer)	31
Kernel size	Low = 2, high = 12 (integer)	11
Number of dense nodes	Low = 1000, high = 5000 (integer)	4927
Dense layer divisor	Low = 0.25, high = 0.8 (real)	0.5653
Dropout	Low = 0, high = 0.5 (real)	0.4860
Batch size	Low = 8, high = 512 (integer)	41

CNN architecture

The hyperparameters, which yielded the best performance, regarding validation loss during the Bayesian optimization process, were used to train the CNN on the combined data of the training set and the validation set. Figure 1 displays a graphical representation of the CNN architecture, while Table S2 provides further details. We trained one-dimensional convolutional kernels since the CNN accepts one-dimensional inputs that cover the complete IR spectrum (intensity gathered at equally-spaced wavenumbers, in 6 cm^{-1} increments across the full 400-4000 cm^{-1} range).

Our CNN architecture, which is a variant of LeNet [5], consists of two convolutional layers for feature extraction and four fully-connected layers for classification. The first convolutional

layer carries out convolutional with a kernel (or filter) size of 11 and stride of 1. The first and second convolution layers have 31 and 62 filters, respectively. We employed padding with zeros evenly to the left and right of the input, resulting in an output that had the same size as the input. Each convolutional layer is followed by a max-pooling layer with a pool size of two, through which a region in the input map is pooled, with a stride of two, into a neuron within the output map. The output of the second max-pooling layer is flattened so that it can be fed into the fully-connected upper layers of the CNN. The first, second, and third dense layers of this CNN have 4927, 2785, and 1574 nodes, respectively. For these upper layers and the convolutional layers, we used the ReLU[6] activation function to introduce nonlinearity between the layers of the network. It is defined as

$$f(x) = \begin{cases} x, & \text{if } x \geq 0 \\ 0, & \text{if } x < 0, \end{cases} \tag{S4}$$

where x is the input to a neuron. The number of outputs in the final dense layer is equal to the number of classes and uses the sigmoid activation function which is defined as

$$\sigma(x) = \frac{1}{1 + e^{-x}}. \tag{S5}$$

Batch normalization[7] was conducted after each convolutional layer and dropout[8] was used after each fully-connected layer to minimize overfitting of the model to the data.

CNN training

The prediction of multi-label functional groups was enabled by employing binary cross-entropy as the loss function, which is defined as

$$J_{bce} = -\frac{1}{M}\sum_{m=1}^{M}\left[y_m \cdot \log\left(h_\theta(x_m)\right) + (1 - y_m) \cdot \log\left(1 - h_\theta(x_m)\right)\right], \quad \text{(S6)}$$

where M is the number of training examples, y_m is the target label for the training example, m, x_m is the input for the training example, m, and h_θ is the model with neural network weights, θ.

The weights of the model were trained using the Adam algorithm[9] for 42 epochs with $\beta_1 = 0.9$, $\beta_2 = 0.999$, and $\epsilon = 1 \times 10^{-8}$. We used a custom learning-rate scheduler to reduce the learning rate at specific epochs. The initial learning rate was 2.5×10^{-4}. At epoch 31, this was reduced to 2.5×10^{-5}. Finally, for epochs 37 to 42 inclusive, the learning rate was reduced to 2.5×10^{-6}. The layers were initialized using a Gaussian distribution with a mean of zero and a variance of 0.05.

We implemented several additional techniques in an attempt to improve the performance of our model, including data augmentation using weighted binary cross-entropy as the loss function, and optimal thresholding.

We augmented the data in four different ways: (1) We introduced random noise that was proportionate to the magnitude at each wavenumber. (2) We translated each spectrum by a few wavenumbers left or right at random. (3) We used a linear combination of a random selection of two spectra for compounds that are described by more than one spectrum in the dataset. The coefficients for each linear combination, which were summed to one, were assigned randomly. (4) We carried out oversampling to act as a control. The amount of data that was augmented was kept constant for all augmentation methods and oversampling: 25%, 50%, 75%, and 100% of the training set and validation set combined. With the exception of the linear combination process, augmentation and oversampling were undertaken on the same selection of samples so that their effects could be compared.

The standard weighted binary cross entropy was employed, as defined by:

$$J_{wbce} = -\frac{1}{M}\sum_{m=1}^{M}\left[w \cdot y_m \cdot \log\left(h_\theta(x_m)\right) + (1 - y_m) \cdot \log\left(1 - h_\theta(x_m)\right)\right], \qquad \text{(S7)}$$

where the weight, w, was implemented to improve our model. An additional weight term was set to adjust the impact of the positive class and give more weight to the minority class.

The optimal threshold for each class was calculated by plotting a precision-recall curve and selecting the corresponding threshold for precision and recall values, which gives the highest F1-score; see section 2.6 for definitions of precision, recall, and F1-score. The performance of our model was evaluated using the calculated optimal thresholds and the default threshold of 0.5 when interpreting the predicted probabilities.

Evaluation metrics

The evaluation metrics used to assess the multi-label classification performance of our model in identifying 37 functional groups are: F1-score, precision, recall, accuracy, average precision, and exact match ratio (EMR). All of these metrics, except for the EMR, were calculated per class label.

Precision represents how many of the returned predictions are true positives and recall measures how many of the true positives are found. The harmonic mean of precision and recall is the F1-score.

We also calculate the accuracy per class label. Rather than calculating the overall accuracy, we separate calculations of accuracy for the presence and the absence of functional groups as there is a large imbalance between the positive class and the negative class.

Furthermore, we report a metric that allows us to focus our attention on the positive class, which we want to find, the average precision (AP). The AP summarizes precision-recall (PR) curves which are sensitive to improvements in the positive class. As we have a heavily imbalanced dataset, where the fraction of the positive class is small compared to that of the negative class, PR curves were useful as a diagnostic tool to aid in the interpretation of probabilistic forecasts for the per-class binary classification. They show the trade-off between the positive predictive value (PPV) and the true positive rate (TPR) for different probability thresholds, allowing us to focus our attention on the positive class. To summarize the PR curves for each target class, the average precision (AP) is calculated as the weighted mean of

precisions that are acquired at each threshold, with the increase in recall from the previous threshold being used as the weight; see Equation S8.

$$AP = \sum_{n} (R_n - R_{n-1}) P_n \,, \tag{S8}$$

where P_n and R_n are the precision and recall at the n-th threshold. The AP provides a better summary when compared to computing the area under the PR curve, which can be too optimistic due to its use of the trapezoidal rule [10].

For a complete analysis, it is paramount to identify all functional groups that are present in a molecule. One way to measure this is by extending the accuracy metric used in single-label cases to multi-label cases. Such an evaluation metric is the EMR, which is the ratio of samples that have all their labels classified correctly; see Equation S9.

$$EMR = \frac{1}{n} \sum_{i=1}^{n} I(Y_i = Z_i) \,, \tag{S9}$$

where n is the number of training examples, Y_i are the true labels for the i-th training example, Z_i are the predicted labels for the i-th training example. It is a harsh metric for multi-label classification performance as it considers all partially correct predictions as incorrect.

Supplementary tables

Table S3. Definition of SMARTS strings and the prevalence of functional groups that were excluded from consideration due to a lack of samples.

Functional group	SMARTS string	Sample frequency
Allene	[CX3]=[CX2]=[CX3]	36
Azide	[$(*-[NX2-]-[NX2+]#[NX1]),$(*-[NX2]=[NX2+]=[NX1-])]	47
Carbamic acid	[NX3][CX3](=[OX1])[OX2H]	1
Cyanate	[OX2][CX2]#N	0
Nitrate	[$([NX3](=[OX1])(=[OX1])O),$([NX3+]([OX1-])(=[OX1])O)]	39
Nitrite	[OX2][NX2]=O	8
Sulfinic acid	[#16X3](=[OX1])[OX2H]	6
Thiocyanate	[SX2][CX2]#N	60
Thioketone	[#6][CX3](=S)[#6]	8

References

(1) National Institute of Advanced Science and Technology, SDBS Web. https://sdbs.db.aist.go.jp (accessed Jan 12, 2021)

(2) Lindstrom, P. J.; Mallard, W. G.; Eds. "Infrared Spectra" in NIST Chemistry WebBook; NIST Standard Reference Database Number 69. National Institute of Standards and Technology: Gaithersburg, MD. https://webbook.nist.gov (accessed Oct 20, 2020).

(3) O'Boyle, N. M. Towards a Universal SMILES Representation - A Standard Method to Generate Canonical SMILES Based on the InChI. *Journal of Cheminformatics* **2012**, *4* (1). https://doi.org/10.1186/1758-2946-4-22.

(4) Landrum, G. RDKit: Open-Source Cheminformatics Software; https://www.rdkit.org. **2016**

(5) Lecun, Y.; Bottou, L.; Bengio, Y.; Haffner, P. Gradient-Based Learning Applied to Document Recognition. *Proceedings of the IEEE* **1998**, *86* (11), 2278–2324. https://doi.org/10.1109/5.726791.

(6) Xu, B.; Wang, N.; Chen, T.; Li, M. Empirical Evaluation of Rectified Activations in Convolutional Network. **2015**. https://doi.org/10.48550/arXiv.1505.00853

(7) Ioffe, S.; Szegedy, C. Batch Normalization: Accelerating Deep Network Training by Reducing Internal Covariate Shift. **2015**. https://doi.org/10.48550/arXiv.1502.03167

(8) Srivastava, N.; Hinton, G. E.; Krizhevsky, A.; Sutskever, I.; Salakhutdinov, R.; Dropout: A Simple Way to Prevent Neural Networks from Overfitting. *Journal of Machine Learning Research* **2014**, *15* (1), 1929-1958.

(9) Kingma, D. P.; Ba, J. Adam: A Method for Stochastic Optimization. **2014**. https://doi.org/10.48550/arXiv.1412.6980

(10) Saito, T.; Rehmsmeier, M. The Precision-Recall Plot Is More Informative than the ROC Plot When Evaluating Binary Classifiers on Imbalanced Datasets. *PLOS ONE* **2015**, *10* (3). https://doi.org/10.1371/journal.pone.0118432.
